# Lateralization of Spatial Relation Processing in Natural Scenes

**DOI:** 10.3233/BEN-2012-129004

**Published:** 2013

**Authors:** Ineke J. M. van der Ham, Martine J. E. van Zandvoort, Albert Postma

**Affiliations:** ^1^Helmholtz InstituteExperimental PsychologyUtrecht UniversityUtrechtThe Netherlands; ^2^Department of NeurologyUniversity Medical Centre UtrechtUtrechtThe Netherlands

**Keywords:** Spatial relations, lateralization, scene perception

## Abstract

Spatial relations between objects can be represented in a categorical and in a coordinate manner. Categorical representations reflect abstract relations, like ‘left of’ or ‘under’, whereas coordinate representations concern exact metric distances between objects. These two types of spatial relations are thought to be linked to a left hemisphere and a right hemisphere advantage, respectively. This lateralization pattern was examined in a visual search task, making use of natural scenes, in patients with unilateral brain damage and healthy controls. In addition, all participants performed a low-level spatial relation processing task. The results suggest that the lateralization pattern commonly found for spatial relation processing in low-level perceptual tasks is also applicable to the processing of complex visual scenes.

## 1. Introduction

Spatial relations between objects can be represented in a categorical and in a coordinate manner. Categorical representations reflect abstract relations, like 'left of' or 'under', whereas coordinate representations concern exact metric distances between objects. These two types of spatial relations are thought to rely on at least partially separate processing mechanisms, reflected in dissociated lateralization patterns. Categorical processing is generally linked to a left hemisphere advantage, while coordinate processing is related to the right hemisphere [1]. The large majority of spatial relation studies have made use of very basic, low-level task designs, using lines, dots and crosses (e.g. [2–4]). However, in the current study, we were interested in seeing whether the lateralization differences still hold in a higher-level, more realistic perceptual task. In particular, we focused on spatial relation processing in stroke patients, to see to what extent lateralized brain damage affects this ability for realistic stimuli. Therefore, we studied visual search performance in stroke patients with either left or right hemisphere damage, and a matched sample of control participants. The main task design entailed natural scenes, i.e. rooms including furniture and other objects. In addition to the visual search task, all participants also performed one of the often used low-level task designs, the cross dot task, to directly compare high-level spatial relation processing to low-level spatial relation processing.

## 2. Method

### 2.1. Participants

Thirty-three patients who suffered from ischaemic or haemorrhagic stroke were included in the experiments. Lesions were located in either the left hemisphere ($N = 16$), or the right hemisphere ($N = 17$). Twenty eight healthy control subjects also participated and were matched to the two patient groups with respect to age and education level.

ISSN 0953-4180/13/$27.50 © 2013 – IOS Press and the authors. All rights reserved

Table 1
Means of all three participant groups on both tasks. LH = left hemisphere damage, RH = right hemisphere damage, Acc = accuracy, RT = response time, cat = categorical, coo = coordinate. Standard deviations in parentheses

| | | Patients | | | | Controls | |
|---|---|---|---|---|---|---|---|
| | | LH | | RH | | | |
| Task | Condition | Acc | RT | Acc | RT | Acc | RT |
| Cross dot | cat large | 89.3 (9.2) | 1229.7 (400.9) | 83.8 (14.5) | 1457.7 (476.0) | 93.2 (9.2) | 1174.1 (173.2) |
| | cat small | 81.2 (18.0) | 1598.0 (827.1) | 80.3 (16.4) | 1528.1 (430.7) | 92.5 (10.1) | 1323.3 (213.8) |
| | coo large | 60.8 (13.8) | 1554.2 (492.4) | 58.6 (11.8) | 1697.6 (345.6) | 68.0 (9.4) | 1322.9 (229.6) |
| | coo small | 74.2 (12.8) | 1474.7 (635.5) | 68.1 (10.7) | 1670.6 (479.2) | 77.8 (9.7) | 1250.1 (237.6) |
| Scenes | coo + cat | 85.8 (13.5) | 10131 (3619) | 80.0 (16.0) | 12028 (5331) | 93.8 (5.4) | 8757 (2205) |
| | coo | 82.7 (15.8) | 9993 (2702) | 74.8 (17.2) | 11683 (3228) | 87.9 (10.4) | 9173 (1911) |

### 2.2. *Cross dot task*

The cross dot task is a match-to-sample working memory task [5]. Participants are presented with a cross combined with a dot. The forty possible dot positions are located in a circular fashion around the cross, at four possible distances. The categorical instruction was to compare both cross dot combinations based on the quadrant of the cross the dots are in, regardless of distance. The coordinate instruction was to compare the two stimuli based on the distance between the dot and the center of the cross, regardless of quadrant. In addition to the two different instructions, two different cross sizes were used. The large cross spanned the entire area where dots could appear, the small cross was only slightly larger than a single dot. The large cross was thought to facilitate categorical processing and the small cross was thought to enhance coordinate processing. Participants performed twenty trials of each of the four instruction * cross size combinations.

### 2.3. *Scenes task*

In a comparative visual search task (see [6]), participants were shown two full-color images of natural scenes, consisting of rooms with furniture and small objects. The two rooms were presented simultaneously, one above the other. The only difference between the two depictions was that one object had moved. Participants needed to detect this change as fast as they could. The position change was labeled either coordinate (coo) or coordinate + categorical (coo + cat). Coordinate change was a change only in distance to the nearest other object. Coordinate + categorical change was a change in both distance and categorical relation (e.g. 'left of' turned into 'above') to the nearest other object. Participants were randomly assigned to one of two versions, to counterbalance for the use of a coordinate change and a coordinate + categorical change in each room. Both change types were matched in the total distance of displacement, so the only cause of a behavioural difference would be the categorical change.

## 3. Results and discussion

In table 1 all means for both tasks are provided for the three participant groups. First of all, the cross dot task showed a clear dissociation between instruction and cross size. A large cross size promoted categorical processing, while coordinate processing benefitted from a small cross size. This suggests that the large cross helps in identifying categories, and obscures the coordinate properties of the stimuli. Analysis of z-scores based on both Acc and RT showed that LH patients were impaired in the coordinate large cross and categorical small cross conditions. RH patients were impaired in both coordinate conditions and in the categorical large cross conditions. Apparently, left hemisphere damage is linked to the ability to construct categories, and right hemisphere damage is linked to coordinate impairment the clearest.

The results of the scene task show that the right hemisphere patient group was impaired in coordinate change detection. In contrast, the left hemisphere patient group was impaired in categorical change detection at trend level. This again suggests a link between coordinate processing and the right hemisphere, and categorical processing and the left hemisphere. Moreover, the control group showed a categorical advantage, indicating that coordinate displacement, as well as categorical changes contribute to location change detection in natural scenes.

A follow-up analysis was performed on both datasets together to study the correlation of the performance on both the low-level and the high-level spatial relation processing tasks. When normalized, there was a highly significant correlation between performance on the low-level cross dot task and the high-level scene task.

This shows a direct link between the two types of relation processing in the commonly used low level setting and the more realistic and complex setting.

## 4. Conclusion

The results suggest that the lateralization pattern commonly found for spatial relation processing in low-level perceptual tasks is also applicable to the processing of complex visual scenes; the left hemisphere is linked to categorical processing and the right hemisphere to coordinate processing. This is substantiated by the fact that performance on spatial relation processing in the low-level cross dot task correlated strongly with performance on the high-level scene task. In addition, it was found that categorical processing benefits from clear categorical boundaries, whereas coordinate processing is better when less visual information is present. In the scene task, it was shown for the healthy control participants that both coordinate and categorical relations are processed when viewing objects in natural scenes, as reflected by the categorical advantage in change detection.
